# *Bacillus subtilis* Promotes Cucumber Growth and Quality under Higher Nutrient Solution by Altering the Rhizospheric Microbial Community

**DOI:** 10.3390/plants12020298

**Published:** 2023-01-08

**Authors:** Bin Li, Lixiang Zhao, Dongxu Liu, Yi Zhang, Wenjiao Wang, Yanxiu Miao, Lingjuan Han

**Affiliations:** 1 College of Horticulture, Shanxi Agricultural University, Jinzhong 030801, China; 2 Quwo Fruit and Vegetable Research Institute, Shanxi Agricultural University, Linfen 043400, China; 3 College of Grassland Science, Shanxi Agricultural University, Jinzhong 030801, China

**Keywords:** *Bacillus subtilis*, rhizospheric microbial community, nutrition solution, cucumber

## Abstract

*Bacillus subtilis* was applied in peat-based soilless cultivation systems containing a mixed substrate (peat:vermiculite:perlite = 2:1:1, *v*/*v*/*v*) and irrigated by one-strength or four-strength Hoagland’s nutrient solution to explore whether it can alleviate inhibition by higher-nutrient solutions (four-strength) and bring benefits to improvements of quality. The results showed that higher-nutrient solutions improved the flavor quality of cucumber fruit; especially, the contents of (E,Z)-2,6-nonadienal and (E)-2-Nonenal were effectively increased, which are the special flavor substances of cucumber. *B. subtilis* K424 effectively improved growth performance, photosynthetic capacity, vitamin C content, soluble sugars, soluble protein, and total pectin in cucumber under higher nutrition solution conditions. Compared with the higher solution treatment, the bacterial diversity significantly increased, whereas the presence of fungi had no significant difference following the *B. subtilis* K424 application. Moreover, *B. subtilis* K424 reduced the relative abundance of *Actinomadura* and promoted that of the *Rhodanobacter*, *Bacillus*, *Pseudomonas*, *Devosiaceae*, and *Blastobotrys* genera. Redundancy analysis showed that *Bacillus*, *Rhodanobacter*, and *Blastobotrys* were positively correlated with the substrate enzyme of sucrase, catalase, and urease. This study provides insight that *B. subtilis* K424 mitigated the deleterious effects of high levels of nutrition solution on cucumber growth and quality by improving the substrate enzyme, regulating the microbial community structure, and enhancing the photosynthetic capacity.

## 1. Introduction

Soilless cultivation, the most efficient and intensive plant production method, is recognized globally in the horticulture industry nowadays [1]. Compared with traditional soil culture, soilless culture has higher yields, efficiency, and quality. The substrate-based soilless culture technology is widely used in China, with the organic substrate containing certain nutrients as the carrier, the cultivation production cost being reduced, and the farming operation being simplified. The advantages are not limited by region, which can greatly expand the agricultural production space, expand the application of noncultivated land, and avoid continuous cropping obstacles in protected cultivation. However, the application of a soilless culture system is closely related to the management of nutrient solutions [2].

Increasing nutrient solution concentration in soilless culture can improve plant quality, but higher salinity in nutrition solutions declined the osmotic ability of environment water and resulted in reduce water uptake by plants, which is beneficial to the accumulation of soluble solids. The saline-nutrient solution applied in hydroponics is a suitable system for sea fennel growth, which gives a slightly salty but high-quality product [3]. The total soluble solid and lycopene concentration of tomato fruits increased with increasing electrical conductivity levels [4]. In addition, salinity led to an imbalance in the nutritional occurrence of oxidative stress and finally decreased plant productivity. Recently, there has been interest in the application of microorganisms in vegetable soilless culture, which could be beneficial to induce plant resistance to biotic and abiotic stress factors and to increase plant growth and yield [5].

Many commercial microbial agents are mainly based on plant growth-promoting rhizobacteria (PGPR), such as *Bacillus* or *Pseudomonas*, which have been used in different countries [6]. The primary mechanisms probably involve the synthesis of various hormones [7,8], increasing the availability of plant nutrients in the rhizosphere [9], and inducing systemic resistance in host plants [10,11]. In addition, they help improve the quality of the soil, such as its physicochemical properties and enzyme activities, which help the growth of rhizosphere microbial communities [12]. The composition of the rhizosphere microbial community is an important factor determining plant health [13], which is also very important for maintaining the healthy and stable microenvironment of plant rhizospheres and successfully relieving various stresses [14].

It is generally believed that higher concentrations of nutrient solutions can improve the impact on the quality of fruit, but it will cause an inhibition of growth. In this study, *B. subtilis* K424 was applied in peat-based soilless cultivation systems which contained a mixed substrate and were irrigated with one-strength or four-strength Hoagland’s nutrient solution, to explore whether it can alleviate the inhibition of growth and bring benefits to the improvement of quality, and to research the potential mechanisms involved in plant tolerance with respect to the composition of microbial communities.

## 2. Results

### 2.1. Growth of Cucumber Seedlings

**Figure 1:**
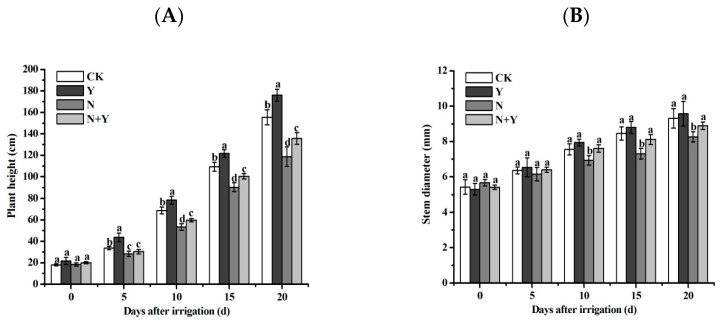
Effects of different treatments on the growth indices of the cucumbers at different time points. (**A**) Plant height; (**B**) stem diameter. Different treatments: CK (control), seedlings were irrigated with a normal nutrient solution; Y, control + *B. subtilis* K424; N, four-strength control; N + Y, four-strength control + *B. subtilis* K424. Each histogram represents the mean ± SE of three independent biological experiments (*n* = 3). Different letters above the bars indicate statistically significant differences by Duncan’s test (*p* < 0.05).

Higher-nutrient solutions (treatment N) seriously decreased the growth of cucumber seedlings, plant height, and stem diameter, which were significantly suppressed compared to the control. However, the *B. subtilis* K424 application had increased effects on the growth of cucumber seedlings under normal and higher-nutrient solutions (Figure 1). Under high nutrient stress, the plant biomass of the cucumber plants pretreated with *B. subtilis* K424 significantly increased after 10 days of irrigation, and the plant growth increased by 1.08–1.14 times compared with untreated plants. It showed that *B. subtilis* K424 application alleviated the inhibition of plant growth induced by high nutrition.

### 2.2. Gas Exchange Parameters

Generally, compared to CK, the leaf gas exchange parameters were significantly increased by the Y treatment, whereas Pn and GS were largely decreased by the N treatment. However, the levels of Pn and Gs were enhanced in the N + Y treatment, indicating that *B. subtilis* K424 can effectively alleviate photosynthetic inhibition caused by high nutrition (Table 1). 

### 2.3. Fruit Qualities in Fresh Cucumbers

To determine whether *B. subtilis* K424 application could effectively change the cucumbers’ qualities, we tested the moisture content, vitamin C, soluble sugar, titratable acidity, sugar/acid, soluble protein, total pectin, soluble solids, and aroma components (Table 2 and Table 3). As shown in Table 2, after the Y treatment, the content of vitamin C and soluble protein improved the most, with values 29.73% and 13.42% higher than the control treatment (CK), respectively. Furthermore, the N treatment significantly increased the content of titratable acidity but decreased the soluble sugar, sugar/acid, vitamin C, soluble protein, and total pectin compared with the N + Y treatment. The highest reduction could be seen for sugar/acid, with a decrease of 31.34%.

As shown in Table 3, a total of 49 volatile components were identified and quantified in different treatments of cucumber: 26 in CK, 29 in the Y treatment, 33 in the N treatment, and 34 in the N + Y treatment. Aldehydes and hydrocarbons were the most abundant compounds in all treatments, representing 54.47–80.59% and 14.39–39.05% of the total content of the volatile components. Compared to the normal nutrient solution-irrigated treatment (CK and Y), the relative contents of (E,Z)-2,6-nonadienal and (E)-2-Nonenal were effectively increased, but there was no significant difference between the treatment of N and N + Y. These results showed that high nutrition application could improve the fruit’s aroma, whereas *B. subtilis* K424 application had no significant effect under high nutrition stress.

### 2.4. Substrate Enzyme Activities

The changes of cucumber growth media enzyme activities including urease, catalase, peroxidase, and sucrase among different treatments were shown in Table 4. Compared to CK, the treatments of Y and N significantly increased the activities of urease and catalase by 46.32–111.18% and 5.26–14.04%, respectively. In addition, compared to the N treatment, the N + Y treatment significantly enhanced the activity of sucrase, but there were no significant differences for the other enzyme activities.

### 2.5. Sequencing and Microbial Diversity Analysis

A total of 802,315 16S rRNA gene sequences and 780,785 fungal ITS sequences were analyzed across the 12 substrate samples, with an average of 66,860 and 65,065 sequences per substrate sample for bacteria and fungi, respectively (Table 5).

**Figure 2:**
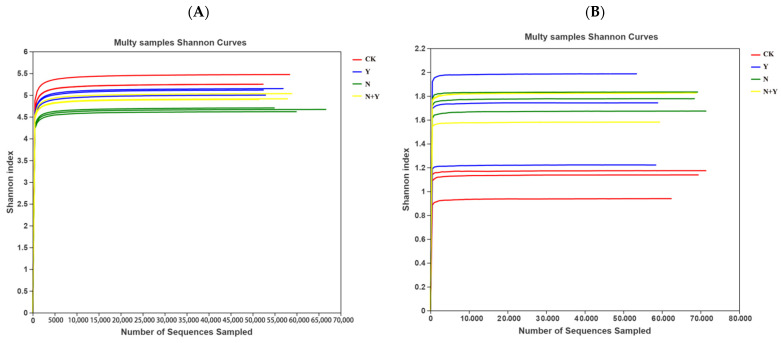
Shannon index curves at 97% similarity levels of rhizospheric growth media samples collected from different treatments. Different treatments: CK (control), seedlings were irrigated with normal nutrient solution; Y, control + *B. subtilis* K424; N, four-strength control; N + Y, four-strength control + *B. subtilis*. K424 (**A**) bacteria; (**B**) fungi.

For all the samples, the Shannon index curves reached near plateaus, indicating that the amount of sequencing data was large enough and the information of the most microbial species had been covered in the samples (Figure 2).

**Figure 3:**
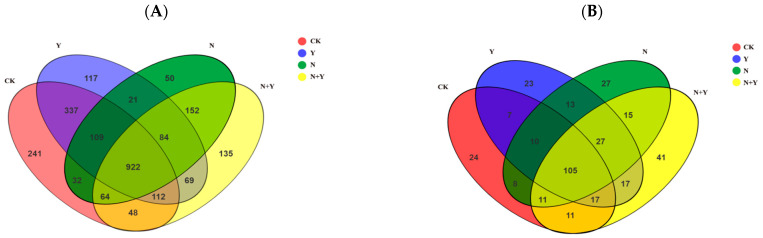
Venn diagrams about unique and shared OTUs of samples collected from different treatments. Different treatments: CK (control), seedlings were irrigated with normal nutrient solution; Y, control + *B. subtilis* K424; N, four-strength control; N + Y, four-strength control + *B. subtilis*. K424 (**A**) bacteria; (**B**) fungi.

Based on 97% species similarity, a total of 2493 OTUs for bacteria and 356 OTUs for fungi were identified, with 922 and 105 OTUs overlapped for bacteria and fungi, respectively (Figure 3). Venn diagrams also showed 241, 117, 50, and 135 unique bacterial OTUs in the CK, Y, N, and N + Y treatments, respectively, whereas 24, 23, 27, and 41 unique OTUs were detected for fungi, respectively.

Alpha diversity analysis showed that the Chao 1 value and ACE in the N + Y-treated soil were generally not significantly different (*p* ≤ 0.05) than those in the N-treated sample, indicating no significant difference in microbial community richness (Table 6). However, the higher Shannon and lower Simpson indexes for bacteria showed that *B. subtilis* K424 increased the bacterial community diversity under high nutrition stress. For fungi, the Shannon and Simpson indexes showed no significantly different results.

### 2.6. Microbial Community Structure

**Figure 4:**
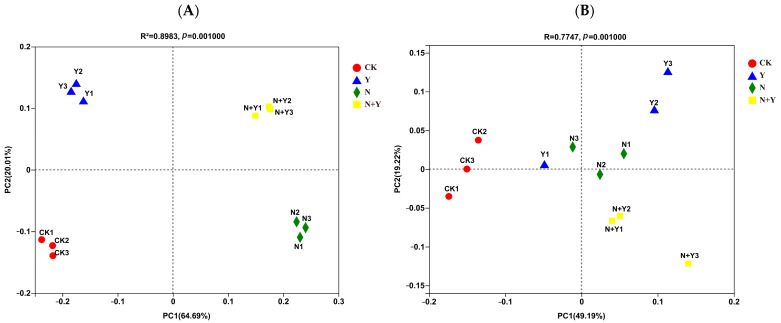
Principal coordinate analysis based on the distance matrix calculated using the Bray–Curtis algorithm for samples collected from different treatments. Different treatments: CK (control), seedlings were irrigated with normal nutrient solution; Y, control + *B. subtilis* K424; N, four-strength control; N + Y, four-strength control + *B. subtilis*. K424 (**A**) bacteria; (**B**) fungi.

A principal coordinate analysis (PCoA) based on the Bray–Curtis algorithm clearly revealed differences in microbial communities across the four treatments (Figure 4). The Bray–Curtis algorithm revealed that all treatments were clearly separated from each other (ANOSIM: R = 1.000, *p* = 0.001 for bacteria; R = 0.775, *p* = 0.001 for fungi). Especially, the Y and N + Y treatments were clearly separated from the other two treatments along the second principal component (PC2) for the bacterial community.

### 2.7. Effects of B. subtilis K424 Application on the Rhizospheric Microbial Community Composition

**Figure 5:**
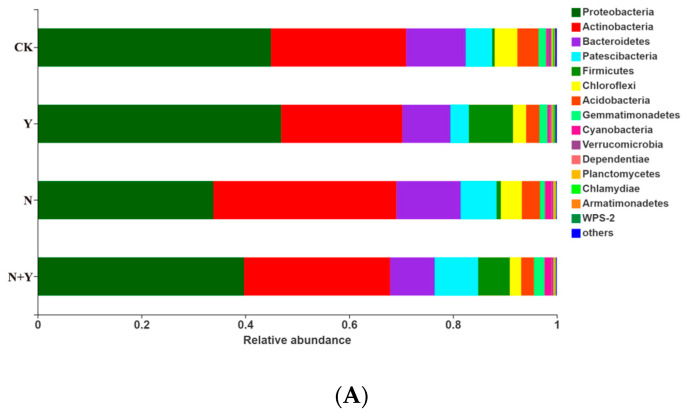

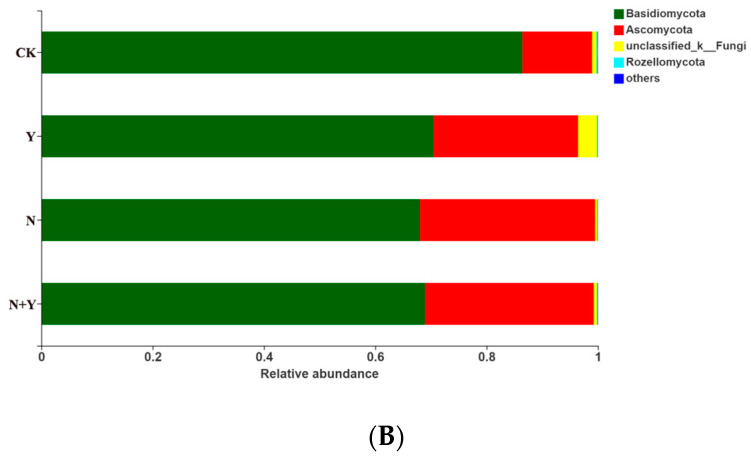
The relative abundance of bacterial (**A**) and fungal phyla (**B**) in samples collected from different treatments. Different treatments: CK (control), seedlings were irrigated with normal nutrient solution; Y, control + *B. subtilis* K424; N, four-strength control; N + Y, four-strength control + *B. subtilis* K424. “Others” indicates phyla with an extremely low abundance (<0.1%).

At the phylum level, 15 bacterial and 3 fungal phyla were identified in all treatments (Figure 5). Of the bacterial phyla, *Proteobacteria*, *Actinobacteria*, *Bacteroidetes*, *Patescibacteria*, and *Firmicutes* were more abundant in all treatments. *B. subtilis* K424 application (Y and N + Y treatments) more greatly enriched the relative abundances of *Proteobacteria* and *Firmicutes* compared with non-inoculation conditions (CK and N treatments). Of the fungal phyla, the Y treatment had a higher relative abundance of *Ascomycota* and lower levels of *Basidiomycota* compared with CK, whereas these showed no significant difference between the N and N + Y treatments.

**Figure 6:**
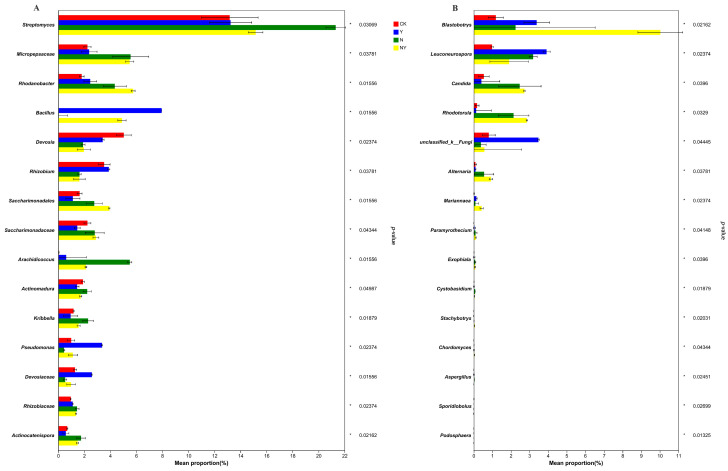
The top 15 classified bacterial (**A**) and fungal genera (**B**) in samples collected from different treatments. Different treatments: CK (control), seedlings were irrigated with normal nutrient solution; Y, control + *B. subtilis* K424; N, four-strength control; N + Y, four-strength control + *B. subtilis* K424.

At the genus level, the analysis of the top 15 classified genera revealed significant differences between different treatments (Figure 6A). Both the Y and N + Y treatments significantly enriched the genera *Rhodanobacter*, *Bacillus*, *Pseudomonas*, and *Devosiaceae* and depleted *Actinomadura* compared with the soil treated with CK and N (false discovery rate (FDR)-adjusted *p* < 0.05, Kruskal–Wallis H test). The relative abundance of the top 15 classified fungal genera (Figure 6B) was not sufficiently enriched in comparison with the bacterial genera. Among the top 15 classified fungal genera, only *Blastobotrys* was significantly more enriched in the Y and N + Y conditions compared with the sample treated with the nutrient solution treatments of CK and N (false discovery rate (FDR)-adjusted *p* < 0.05, Kruskal–Wallis H test).

### 2.8. Correlation between Rhizospheric Microbiota and Substrate Indexes

**Figure 7:**
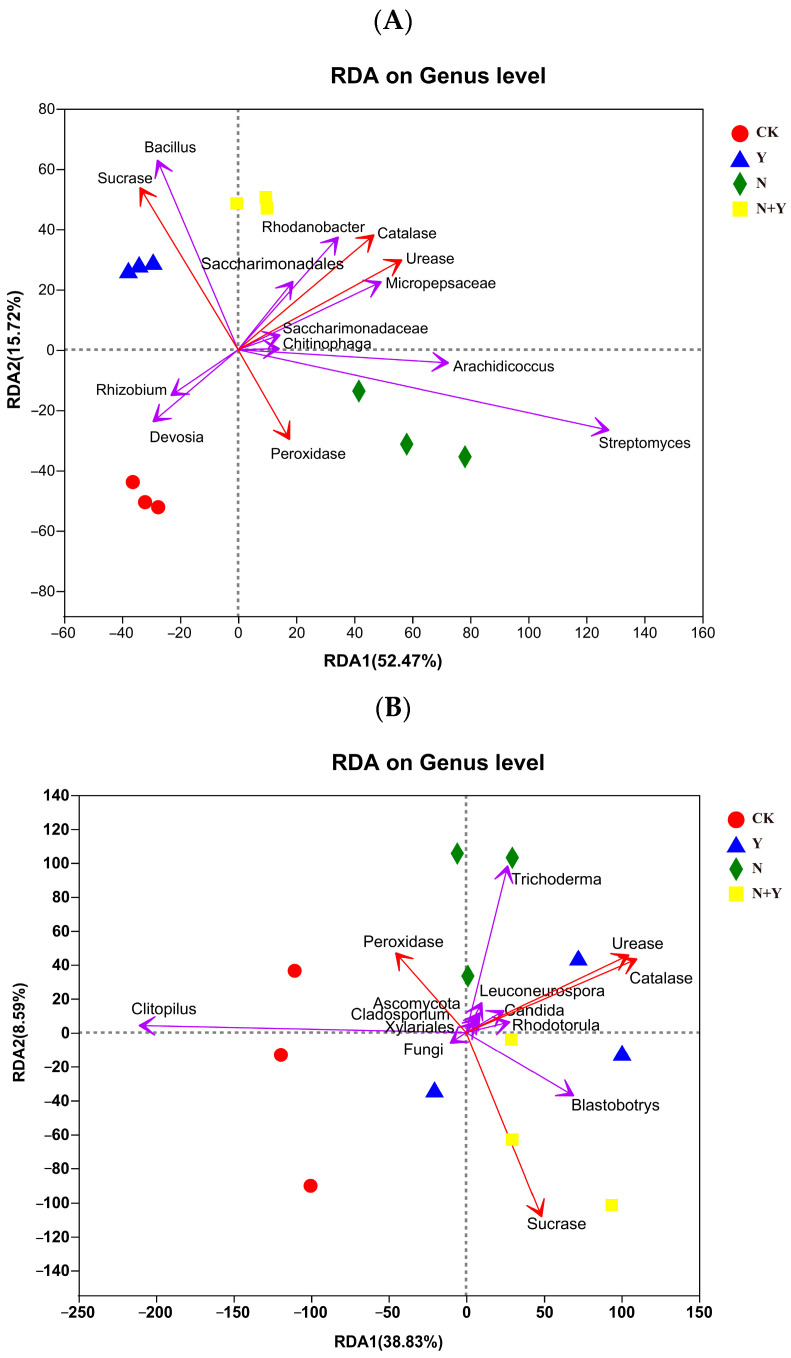
Redundancy analysis (RDA) based on the OTUs of the top 10 bacterial (**A**) and fungal (**B**) genera and selected environmental variables for samples collected from different treatments. Different treatments: CK (control), seedlings were irrigated with normal nutrient solution; Y, control + *B. subtilis* K424; N, four-strength control; N + Y, four-strength control+ *B. subtilis* K424.

A Mantel test based on the selected indexes of substrate and top 10 bacterial or fungal genera abundance revealed a significant correlation in the microbial communities (Bray–Curtis distance, r = 0.821, *p* = 0.001 for bacteria and r = 0.522, *p* = 0.005 for fungi). The RDA analysis showed that the first and second RDA components were able to explain 68.19% and 47.42% of the total bacterial and fungal communities, respectively (Figure 7). As shown by their close grouping and vectors, *Bacillus* and *Rhodanobacter*, which had a higher relative abundance upon Y and N + Y treatment, displayed strong associations with sucrase, catalase, and urease. While the fungal genus, the abundance of *Blastobotrys* was positively correlated with sucrase, catalase, and urease.

## 3. Discussion

In this study, we found that *B. subtilis* K424 application had a positive effect on the growth of cucumber plants, fruit quality, and photosynthesis, suggesting that *B. subtilis* K424 had the capacity to alleviate the toxic effects of salinity stress (higher-strength nutrition) on the plant growth and photosynthetic performance. The previous studies showed that low nutrient concentrations improved micronutrient nutrition and water use efficiency in soilless culture systems, and might be sufficient for the plant’s normal growth [15,16]. PGPR has a direct growth-promoting effect on plants, which can alleviate the damage of plants to various stresses. [17]. The fruit quality results showed that the four-strength nutrient solution increased the titratable acidity and soluble solids of the cucumber. This is in line with previous findings that titratable acidity and soluble solids will increase with salinity [18]. In addition, aldehyde compounds, particularly (E,Z)-2,6-nonadienal and (E)-2-Nonenal, have a powerful fresh cucumber odor impact [19]. Nevertheless, our results showed no significant effect on the higher-strength nutrition stress after *B. subtilis* K424 application. In the current study, *B. subtilis* K424 markedly improved the photosynthetic rate and stomatal conductance under saline stress (Table 1). Consistently, Li indicated that the maize plant photosynthesis rate and stomatal conductance under salinity stress were increased by PGPR strain [20]. Moreover, salinity can significantly reduce the gaseous exchange and total chlorophyll contents. However, the mechanism underlying the stimulation of plant photosynthesis by PGPR remains elusive [21].

Our research was carried out to explore how *B. subtilis* K424 application influences variations in the substrate’s enzymatic activities and rhizosphere microbiome to alleviate salinity stress. Many reports show that the plant rhizosphere can recruit many microbial components when they are attacked by biotic and abiotic stress, such as pathogens [14] and heavy metal and salt stresses [22,23]. Therefore, the application of *B. subtilis* K424 in our study may have an impact on the recruitment of bacteria and fungi in the cucumber rhizosphere, thereby altering various aspects of the substrate environment. Xiong et al. [24] reported that after the application of microbiological agents, the soil health was improved by the direct suppression of pathogens or via the modification of the indigenous microbial community. Compared to the N-treated sample, *B. subtilis* K424 application (N + Y-treated) significantly increased the Shannon index and decreased the Simpson index for bacteria, whereas those showed no significant difference for fungi (Table 6). This implied that *B. subtilis* K424 inoculation mainly helps to restore the rhizospheric bacterial diversity negatively affected by higher-strength nutrition. Similar to our study, soil microbial bacterial abundance or higher diversity in communities favored the ability of plants to suppress adverse environments [25,26].

Microbial diversity analysis showed that the application of *B. subtilis* K424 altered bacterial and fungal communities (Figure 3), consistent with previous observations that biofertilizer and PGPR inoculation generally resulted in different soil microbial community structures [14,27]. *Actinobacteria* was the most abundant bacterial phyla identified in the N treatment soil, in parallel with the outcome of a previous study investigating the dominant microbial phylum in saline ecosystems [28]. The phyla *Proteobacteria* and *Firmicutes* were more abundant in *B. subtilis* K424-applied soil, and these two phyla have evolved salt stress-tolerant genetic machinery to adapt and survive under salt-induced osmotic stresses [29,30]. Previous studies have also found higher abundances of *Proteobacteria* and *Firmicutes* in biofertilizer-treated soils [31,32]. Two fungal phyla, *Ascomycota* and *Basidiomycota*, which occupy more than 90% of the total fungal sequence, were the most dominant phyla across all samples. However, these showed no significant difference between the N and N + Y treatments. These indicated that there was a much weaker rhizosphere effect on the fungal communities than on the bacterial communities by the *B. subtilis* K424 application under salt stress. This is in line with our previous findings that *B. subtilis* K424 significantly increased the bacterial community diversity, whereas the presence of the fungi caused no changes under salt stress.

Genera abundance analysis showed that *B. subtilis* K424 increased the abundance of *Rhodanobacter*, *Bacillus*, *Pseudomonas*, *Devosiaceae*, and *Blastobotrys* in the rhizosphere samples (Figure 6). Previous studies have shown that *Rhodobacter*, *Bacillus*, and *Pseudomonas* promote plant growth even under adverse environmental conditions [29,33]. In addition, the *Pseudomonas* species and its ability to produce exopolysaccharides (EPS) enhance tolerance to salt stress [34]. *Bacillus*, which produces higher expressions of antioxidant enzymes, may lead to plant cell protection in stress conditions [29,35]. In our research, the enrichment of *Bacillus* was probably due to the addition of *B. subtilis* K424. However, due to the analysis technology limitation of pyrosequencing, the information about the strain can only be classified at the genera level. RDA analysis revealed a correlation between the environmental variables and the top 10 abundant genera. The Y and N + Y treatments were dominated by *Bacillus*, *Rhodanobacter*, and *Blastobotrys*, which were positively correlated with sucrase, catalase, and urease. This result was similar to that of Shi et al. [14], who found that the PGPR strain, NSY50, maintained an optimal environment to alleviate stress by altering soil enzyme activities, phy-chemical properties, and microbial communities. However, the mechanisms have been poorly investigated for their complex interaction between soil properties and the microbial community.

## 4. Materials and Methods

### 4.1. Pot Experiment Design

The pot experiment was carried out in Shanxi Agricultural University, China. Cucumber seeds (*Cucumis sativus* L. cv. Jinchun No. 2) were surface-sterilized in 70% ethanol for 30 s, followed by 20 min of shaking in a 2% sodium hypochlorite solution, washed in sterile water three times, and placed in 10cm Petri dishes covered with sterile wet filter paper for germination in the dark at 28 °C for 24 h. Germinated seeds were transferred into quartz sand and cultivated in a greenhouse with day/night temperatures of 25–30 °C/15–18 °C and relative humidity of 60–72%. At the stage of two true leaves, the plants were transferred into pots and treated with nutrient solution after 3 days.

A pot experiment was performed using a randomized complete block design with three replicates for each treatment, where each replicate had sixteen plants, and each pot (diameter, 250 mm; volume 3200 mL) had one plant contained a mixed substrate (peat: vermiculite: perlite = 2:1:1, *v*/*v*/*v*).

Four treatments were designed as follows: (1) CK, untreated substrate without *B. subtilis* K424, and the plants were irrigated with one-strength Hoagland’s nutrient solution (Ca(NO_3_)_2_·4H_2_O 945 mg/L, KNO_3_ 607 mg/L, NH_4_H_2_PO_4_ 115 mg/L, MgSO_4_·7H_2_O 493 mg/L, EDTA-NaFe 30 mg/L, H_3_BO_3_ 2.86mg/L, MnSO_4_·4H_2_O 2.13 mg/L, ZnSO_4_·7H_2_O 0.22 mg/L, CuSO_4_·5H_2_O 0.08 mg/L, (NH_4_)_6_Mo_7_O_24_·4H_2_O 0.02mg/L), (2) Y, treated substrate with *B. subtilis* K424, and the plants were irrigated one-strength Hoagland’s nutrient solution, (3) N, untreated substrate without *B. subtilis* K424, and the plants were irrigated four-strength Hoagland’s nutrient solution, and (4) N + Y, treated substrate with *B. subtilis* K424, and the plants were irrigated with four-strength Hoagland’s nutrient solution. The *B. subtilis* K424, isolated from the greenhouse soil by our laboratory at Shanxi Agricultural University, was added to and thoroughly mixed with the substrate before planting, which contained 10^8^ cfu/g. Each pot was supplemented with 1.5 g *B. subtilis* K424. The whole growth process was supplemented with an equivalent volume nutrient solution 5 times every week, and the growth period lasted for 60 days.

### 4.2. Assays of Growth Indices

The plant heights and stem diameters were recorded manually every 5 days after the first supplementation of the nutrient solution. Three seedlings were randomly selected from each treatment with three replications.

### 4.3. Determination of Leaf Photosynthetic Parameters

The gas exchange parameters of the fully expanded leaves, which were the third leaves from the base of the stem, were measured using a portable photosynthesis system (LI-6400, LI-COR Inc., Lincoln, NE, USA), with the light intensity, external CO_2_ concentration, leaf temperatures, and RH in the assimilation chamber maintained at 1000 µmol photons m^−2^ s^−1^, 380 ± 10 µmol mol^−1^, 25 °C, and 70%, respectively. The photosynthetic parameters of photosynthetic rate (Pn), stomatal conductance (Gs), intercellular CO_2_ concentration (Ci), and transpiration rate (Tr) were measured ten days after nutrient solution treatment.

### 4.4. Determination of Qualities in Fresh Cucumbers

Cucumbers reaching commercial maturity standards of about 30 cm were harvested. In the full fruit period, the cucumber fruits with consistent growth, and the same nodes were picked to determine the related quality and fruit aroma indicators.

The moisture content of the fruit was determined with direct drying in a 45 °C oven. The vitamin C content was analyzed using the method of Gül et al. [5]. The titratable acidity was determined using 0.1 mol·L^−1^ NaOH to neutralize all the titratable protons up to pH 8.1. The soluble protein was determined using the method of Bradford [36]. The soluble sugar was analyzed using the method of Blakeney and Mutton [37]. The total soluble solid was determined using an automatic temperature-compensated digital refractometer (Atago Pallette PR101, Atago Co., Tokyo, Japan). The total pectin was assayed by the Carbazole colorimetry method [38].

Solid phase micro-extraction and gas chromatography–mass spectrometry (GC–MS) were used to explore the aroma components of the cucumber fruits. Cucumbers without peel (exocarp tissue) were blended for 30 s using a mechanical blender just before extraction. Immediately after blending, a 10 mL sample paste was placed into a 15 mL sealed headspace vial. The samples were continuously extracted in a 40 °C water bath for 40 min. After extraction, the fiber was manually removed from the vial and immediately inserted into the GC–MS injector for analysis.

A 7890A GC system (Agilent Technologies, Santa Clara, CA, USA) combined with a 5975C MSD (Agilent Technologies) was used for GC–MS analysis. The chromatographic column was an HP-INNWAX (60 m × 0.25 mm × 0.25 µm), with high-purity helium as the gas carrier, at a flow rate of 1.0 mL/min. The injector temperature was 220 °C, and it was equipped with a splitless injector. The temperature was set initially to 40 °C (held for 2.5 min) and increased to 230 °C at 6 °C/ min (held for 7 min). The MS ion source temperature was 250 °C, and the electron energy was 70 eV. The scan range was 35–400 amu, the emission current was 100 µA, and the detection voltage was 1.4 kV.

### 4.5. Analysis of Substrate Enzyme Activity and DNA Extraction

The rhizosphere substrate samples were obtained from the three biological replicate pots after harvesting the fruits, and each collected sample (sieved 2 mm) was divided into two portions. One portion was air-dried for evaluating the soil enzyme activity, and the other portion was frozen at −80 °C for DNA extraction.

The substrate urease activity was analyzed using the method of Kandeler and Gerber [39]. The catalase activity was determined according the method of Xue and Huang [40]. The methods of Ge et al. [41] and Bartha and Bordeleau [42] were used to assay the sucrase and peroxidase activity, respectively.

Total DNA was extracted from 0.5 g of each fresh substrate sample using a Power soil DNA Extraction kit (MOBIO Laboratories, Carlsbad, CA, USA) following the manufacturer’s instructions. The concentration and quality of the extracted DNA were quantified with an Eppendorf Biophotometer plus (Eppendorf, Germany), and the DNA was stored at −20 °C.

### 4.6. PCR Amplification and Miseq Sequencing

The bacterial community composition was assessed by sequencing the V3-V4 region of the 16S rRNA using the primers 338F(5′-ACTCCTACGGGAGGCAGCAG-3′) and 806R(5′-GGACTACHVGGGTWTCTAAT-3′). The fungal ITS region was amplified using the primer sets ITS1 (5′-CTTGGTCATTTAGAGGAAGTAA-3′) and ITS2(5′-GCTGCGTTCTTCATCGATGC-3′).

The PCR reactions were performed in a 20 μL reaction mixture containing 4 μL of 5 × FastPfu Buffer, 2 μL of 2.5 mM dNTPs, 0.8 μL of each primer (5 μM), 0.4 μL of FastPfu Polymerase, 0.2 μL of BSA, and 10 ng of template DNA. The PCR reactions were conducted using the following program: initiation at 95 °C for 3 min, 30 (bacteria) or 36 (fungi) cycles of denaturation at 95 °C for 30 s, annealing at 55 °C for 30 s, and extension at 72 °C for 45 s, and a final elongation at 72 °C for 10 min. The samples were checked and extracted from a 2% agarose gel, and then further purified using a PCR purification kit (Axygen Biosciences, Union City, CA, USA). The PCR products were quantified using QuantiFluor™-ST (Promega, Madison, WI, USA) according to the manufacturer’s protocol.

High-throughput sequencing was performed on the Illumina MiSeq sequencer at Majorbio Bio-pharm Technology Co., Ltd., (Shanghai, China). Briefly, the raw sequences were processed using QIIME (version 1.9.1). The operational taxonomic units (OTUs) were clustered using UPARSE (version 7.0.1090) with a 97% similarity threshold. According to OTUs information, a series of in-depth visual analyses were performed, such as the community composition of multiple samples, multivariate analysis, and a difference significance test.

### 4.7. Statistical Analyses

SPSS 20.0 program (SPSS Inc., Chicago, IL, USA) was used to statistically analyze the data. The significance between the treatments was assessed by one-way analysis of variance (ANOVA) using Duncan’s test.

According to the NIST08.L MS data library, the chromatographic peaks were analyzed and identified. The relative content of volatile matter was calculated by the formula:

Relative content (%) = single constituent area/total area × 100%

The venn diagrams and Alpha-diversity (ACE, Chao1, Shannon, and Simpson index) indices were calculated according to the OTU assignment using Mothur software (version v.1.30.2. https://mothur.org/wiki/calculators/, accessed on 1 January 2023). The ACE and Chao1 indexes measured the species richness and the number of species. Shannon and Simpson indexes were used to measure species diversity, which was affected by the species richness and the community evenness in the sample community. Principal Coordinate Analysis (PCoA) was performed to evaluate beta-diversities between the four treatments. The significant differences in the community structures were examined by analysis of similarity (ANOSIM). Redundancy analysis (RDA) was conducted using Canoco version 4.5 (Biometry, Wageningen, The Netherlands).

## 5. Conclusions

In conclusion, the results from the present study demonstrate that the application of *B. subtilis* K424 alleviates higher-strength nutrition stress by changing the substrate enzyme and microbial community structure, thus increasing the beneficial microbes such as *Rhodanobacter*, *Bacillus*, and *Pseudomonas*. Ultimately, this produces an optimal condition to stimulate plant growth and fruit quality.

## Figures and Tables

**Table 1:** Photosynthetic characteristics of cucumber leaves under different treatments.

Treatment	P_n_ (µmol CO_2_ m^−2^ s^−1^)	G_S_ (mmol H_2_O m^−2^ s^−1^)	C_i_ (µmol mol^−1^)	T_r_ (mmol m^−2^ s^−1^)
CK	20.84 ± 0.32 b	0.39 ± 0.04 b	320.36 ± 14.43 a	4.53 ± 0.18 c
Y	26.25 ± 0.79 a	0.68 ± 0.03 a	313.46 ± 15.59 a	7.49 ± 0.13 a
N	16.11 ± 0.14 d	0.28 ± 0.02 c	297.83 ± 10.94 a	6.06 ± 0.16 b
N + Y	19.45 ± 0.29 c	0.36 ± 0.02 b	295.16 ± 10.27 a	6.38 ± 0.22 b

Note: P_n_, Note: Pn, photosynthetic rate; G_s_, stomatal conductance; C_i_, intercellular CO_2_ concentration; T_r_, transpiration rate. The third leaves of the plants at 10 days after the beginning of the treatment were used to measure photosynthetic capacity. Values represent the mean ± SE (*n* = 3). Different letters indicate significant differences at *p* < 0.05 according to Duncan’s multiple range tests. Different treatments: CK (control), seedlings were irrigated with normal nutrient solution; Y, control + *B. subtilis* K424; N, four-strength control; N + Y, four-strength control + *B. subtilis* K424.

**Table 2:** Fruit qualities of fresh cucumbers under different treatments.

Treatment	Moisture Content (%)	Vitamin C (μg·g^−1^)	Soluble Sugar (μg·g^−1^)	Titratable Acidity (μg·g^−1^)	Sugar/Acid	Soluble Protein (μg·g^−1^)	Total Pectin (μg·g^−1^)	Soluble Solids (μg·g^−1^)
CK	95.52 ± 0.19 b	117.98 ± 5.93 c	15.86 ± 0.14 b	0.75 ± 0.03 bc	21.16 ± 0.09 c	10.58 ± 0.38 d	54.81 ± 1.89 a	37.36 ± 3.69 b
Y	96.02 ± 0.00 a	153.05 ± 6.94 b	17.43 ± 0.20 a	0.79 ± 0.01 ab	22.14 ± 0.41 b	12.00 ± 0.17 c	54.19 ± 3.60 a	27.75 ± 4.49 c
N	95.10 ± 0.16 c	143.25 ± 0.61 b	16.12 ± 0.20 b	0.83 ± 0.06 a	19.40 ± 1.55 c	13.11 ± 0.37 b	46.38 ± 5.32 b	47.61 ± 8.38 a
N + Y	94.76 ± 0.04 c	175.41 ± 6.52 a	17.57 ± 0.24 a	0.69 ± 0.01 c	25.48 ± 0.25 a	13.88 ± 0.30 a	60.77 ± 3.02 a	48.35 ± 3.44 a

Note: Values represent the mean ± SE (*n* = 3). Different letters indicate significant differences at *p* < 0.05 according to Duncan’s multiple range tests. Different treatments: CK (control), seedlings were irrigated with normal nutrient solution; Y, control + *B. subtilis* K424; N, four-strength control; N + Y, four-strength control + *B. subtilis* K424.

**Table 3:** The relative contents and aroma compounds of fresh cucumbers in different treatments.

NO.	Compound	Relative Percentage Content (%)			
		CK	Y	N	N + Y
	Aldehydes				
1	Hexanal	4.40 ± 0.12 a	3.37 ± 0.21 b	1.13 ± 0.22 d	2.11 ± 0.20 c
2	2-Hexenal	1.72 ± 0.10 a	1.89 ± 0.04 a	0.47 ± 0.20 b	0.55 ± 0.20 b
3	Heptanal	0.28 ± 0.06 a	0.24 ± 0.02 a	0.00 ± 0.00 b	0.30 ± 0.12 a
4	2-Nonenal, (E)	25.61 ± 1.08 b	20.79 ± 2.17 c	35.25 ± 1.50 a	35.96 ± 1.82 a
5	2-Nonenal, (Z)	0.00 ± 0.00 b	0.00 ± 0.00 b	0.00 ± 0.00 b	0.21 ± 0.02 a
6	Nonanal	22.32 ± 1.40 a	15.21 ± 0.57 b	11.98 ± 1.42 c	12.56 ± 0.30 c
7	2,6-Nonadienal, (E,Z)	19.16 ± 0.83 b	12.81 ± 0.30 c	31.52 ± 5.01 a	28.75 ± 0.40 a
8	Decanal	0.24 ± 0.08 a	0.00 ± 0.00 b	0.00 ± 0.00 b	0.00 ± 0.00 b
9	1-Cyclohexene-1-carboxaldehyde, 2,6,6-trimethyl	0.00 ± 0.00 c	0.16 ± 0.03 a	0.09 ± 0.02 b	0.15 ± 0.03 a
	Alcohols				
10	1-Penten-3-ol	0.00 ± 0.00 c	0.00 ± 0.00 c	0.13 ± 0.01 b	0.25 ± 0.10 a
11	1-Hexanol	0.28 ± 0.02 a	0.00 ± 0.00 c	0.00 ± 0.00 c	0.17 ± 0.03 b
12	Eucalyptol	0.00 ± 0.00 b	0.00 ± 0.00 b	0.06 ± 0.01 a	0.06 ± 0.01 a
13	trans,cis-2,6-Nonadien-1-ol	0.00 ± 0.00 b	0.00 ± 0.00 b	0.77 ± 0.05 a	0.00 ± 0.00 b
14	2-Nonen-1-ol	0.00 ± 0.00 b	0.00 ± 0.00 b	1.43 ± 0.16 a	0.00 ± 0.00 b
15	3-Decyn-2-ol	0.07 ± 0.02 a	0.00 ± 0.00 b	0.00 ± 0.00 b	0.06 ± 0.02 a
16	trans-3-nonen-1-ol	0.28 ± 0.02 a	0.16 ± 0.06 ab	0.13 ± 0.02 b	0.21 ± 1.14 a b
17	n-Tridecan-1-ol	0.38 ± 0.05 a	0.28 ± 0.02 a	0.36 ± 0.18 a	0.21 ± 0.22 a
	Ketones				
18	Ethanone, 1-(2-methyl-1-cyclopenten-1-yl)	0.16 ± 0.04 bc	0.20 ± 0.03 b	0.13 ± 0.04 c	0.27 ± 0.02 a
19	Cyclohexanone, 2,2,6-trimethyl	0.00 ± 0.00 b	0.00 ± 0.00 b	0.05 ± 0.00 a	0.06 ± 0.00 a
	Hydrocarbons				
20	Heptane	0.28 ± 0.03 a	0.36 ± 0.08 a	0.00 ± 0.00 b	0.00 ± 0.00 b
21	Di-tert-butyl peroxide	7.98 ± 0.19 b	19.71 ± 1.40 a	3.10 ± 0.13 c	3.99 ± 0.46 c
22	Cyclobutene, 2-propenylidene	0.00 ± 0.00 b	0.00 ± 0.00 b	0.09 ± 0.01 a	0.00 ± 0.00 b
23	Toluene	0.00 ± 0.00 b	0.00 ± 0.00 b	0.00 ± 0.00 b	0.13 ± 0.02 a
24	2-Octene	0.00 ± 0.00 b	0.04 ± 0.00 a	0.02 ± 0.02 a	0.04 ± 0.01 a
25	3-Octyne	0.00 ± 0.00 b	0.00 ± 0.00 b	0.00 ± 0.00 b	0.02 ± 0.02 a
26	Cyclopentane, propyl	1.25 ± 0.06 a	1.12 ± 0.10 a	0.39 ± 0.17 c	0.68 ± 0.04 b
27	2,3,5-trimethylhexa-1,3-diene	0.00 ± 0.00 b	0.00 ± 0.00 b	0.03 ± 0.00 a	0.00 ± 0.00 b
28	1,2,4,4-Tetramethylcyclopentene	0.00 ± 0.00 b	0.00 ± 0.00 b	0.00 ± 0.00 b	0.02 ± 0.00 a
29	1-hydroperoxyhexane	0.00 ± 0.00 b	0.00 ± 0.00 b	0.13 ± 0.02 a	0.00 ± 0.00 b
30	p-Xylene	0.07 ± 0.04 a	0.00 ± 0.00 b	0.00 ± 0.00 b	0.00 ± 0.00 b
31	Nonane	1.08 ± 0.23 a	1.24 ± 0.49 a	0.31 ± 0.11 b	0.42 ± 0.04 b
32	à-Pinene	0.00 ± 0.00 b	0.20 ± 0.03 a	0.00 ± 0.00 b	0.00 ± 0.00 b
33	á-Phellandrene	0.05 ± 0.01 a	0.00 ± 0.00 b	0.00 ± 0.00 b	0.00 ± 0.00 b
34	Bicyclo [3.1.0]hexane, 4-methylene-1-(1-methylethyl)	0.00 ± 0.00 cb	0.2 ± 0.01 a	0.03 ± 0.01 b	0.02 ± 0.01 b
35	o-Cymene	0.00 ± 0.00 b	0.08 ± 0.01 a	0.00 ± 0.00 b	0.00 ± 0.00 b
36	1,6-Dioxaspiro[4.4]nonane, 2-ethyl	0.00 ± 0.00 b	0.00 ± 0.00 b	0.14 ± 0.02 b	0.25 ± 0.02 a
37	Undecane	0.00 ± 0.00 b	0.00 ± 0.00 b	0.02 ± 0.00 a	0.00 ± 0.00 b
38	1-Nonene	2.99 ± 0.21 b	3.17 ± 0.08 b	5.83 ± 1.41 a	4.47 ± 1.57 ab
39	1-Tridecene	0.09 ± 0.02 a	0.00 ± 0.00 b	0.00 ± 0.00 b	0.00 ± 0.00 b
40	Tridecane	0.19 ± 0.05 a	0.04 ± 0.00 b	0.14 ± 0.04 a	0.23 ± 0.04 a
41	Tetradecane	0.00 ± 0.00 b	0.36 ± 0.05 a	0.42 ± 0.04 a	1.01 ± 0.28 a
42	Bicyclo[7.2.0]undec-4-ene, 4,11,11-trimethyl-8-methylene-,[1R-(1R*,4Z,9S*)]	0.24 ± 0.03 a	0.24 ± 0.01 a	0.08 ± 0.02 b	0.00 ± 0.00 b
43	Caryophyllene	5.16 ± 0.11 b	7.55 ± 0.28 a	2.51 ± 1.42 c	1.27 ± 0.01 c
44	trans-à-Bergamotene	0.16 ± 0.03 a	0.00 ± 0.00 b	0.00 ± 0.00 b	0.25 ± 0.12 a
45	Bicyclo[3.1.1]hept-2-ene, 2,6-dimethyl-6-(4-methyl-3-pentenyl)	0.00 ± 0.00 c	0.20 ± 0.02 a	0.14 ± 0.01 b	0.15 ± 0.01 b
46	Humulene	2.85 ± 0.07 b	4.54 ± 0.29 a	1.57 ± 0.23 c	1.44 ± 0.41 c
47	Others				
	Furan, 2-ethyl	0.00 ± 0.00 d	0.92 ± 0.04 a	0.39 ± 0.11 c	0.59 ± 0.06 b
48	Bicyclo[3.1.0]hex-2-ene, 4-methyl-1-(1-methylethyl)	0.00 ± 0.00 b	0.08 ± 0.02 a	0.00 ± 0.00 b	0.00 ± 0.00 b
49	Furan, 2-pentyl	2.71 ± 0.64 ab	2.45 ± 0.21 b	1.21 ± 0.08 c	3.12 ± 0.14 a

Note: Values represent the mean ± SE (*n* = 3). Different letters indicate significant differences at *p* < 0.05 according to Duncan’s multiple range tests. * represent the chiral center on the substituent outside the parent structure. Different treatments: CK (control), seedlings were irrigated with normal nutrient solution; Y, control + *B. subtilis* K424; N, four-strength control; N + Y, four-strength control + *B. subtilis* K424.

**Table 4:** Effects of cucumber rhizospheric growth media enzyme activities in different treatments.

Treatment	Urease (mg/g·h^−1^)	Catalase (mg/g·h^−1^)	Peroxidase (μg/g·h^−1^)	Sucrase (mg/g·h^−1^)
CK	19.41 ± 0.48 c	0.57 ± 0.02 c	63.93 ± 3.97 a	3.75 ± 0.03 b
Y	28.40 ± 0.64 b	0.60 ± 0.00 b	63.57 ± 6.37 a	4.05 ± 0.36 b
N	40.99 ± 1.09 a	0.65 ± 0.02 a	66.78 ± 3.75 a	2.93 ± 0.20 c
N + Y	37.67 ± 3.29 a	0.66 ± 0.02 a	59.33 ± 2.76 a	4.84 ± 0.25 a

Note: Values represent the mean ± SE (*n* = 3). Different letters indicate significant differences at *p* < 0.05 according to Duncan’s multiple range tests. Different treatments: CK (control), seedlings were irrigated with normal nutrient solution; Y, control + *B. subtilis* K424; N, four-strength control; N + Y, four-strength control + *B. subtilis* K424.

**Table 5:** Retained sequences that were used for further analysis after removing the short, ambiguous, and low-quality reads of the rhizospheric samples collected from different treatments.

Sample	Retained Sequences	
	Bacterial 16S rRNA Genes	Fungal ITS Sequences
CK1	60,766	62,970
CK2	67,166	72,769
CK3	61,550	70,545
Y1	69,043	58,851
Y2	72,138	59,491
Y3	66,874	54,379
N1	72,269	70,028
N2	64,099	69,633
N3	74,765	71,773
N + Y1	56,886	59,880
N + Y2	66,420	69,166
N + Y3	70,339	61,300
Mean	66,860	65,065
Total	802,315	780,785

Note: CK (control), seedlings were irrigated with normal nutrient solution; Y, control + *B. subtilis* K424; N, four-strength control; N + Y, four-strength control + *B. subtilis*. K424.

**Table 6:** The diversity and richness estimates for the different rhizosphere samples at 97% similarity.

Treatment	Community Characteristics							
	Bacterial Community				Fungal Community			
	ACE	Chao1	Shannon	Simpson	ACE	Chao1	Shannon	Simpson
CK	1732.92 a	1705.46 a	5.32 a	0.01 c	172.68 a	162.71 a	1.08 b	0.61 a
Y	1634.19 a b	1648.51 ab	5.09 b	0.02 b	193.60 a	185.89 a	1.65 a	0.42 b
N	1370.63 c	1388.97 c	4.66 c	0.03 a	203.21 a	190.51 a	1.76 a	0.37 b
N + Y	1508.23 b c	1494.90 b c	4.95 b	0.02 b	218.91 a	200.32 a	1.74 a	0.39 b

Note: Different letters in each column indicate statistically significant differences based on Duncan’s test (*p* < 0.05). CK (control), seedlings were irrigated with normal nutrient solution; Y, control + *B. subtilis* K424; N, four-strength control; N + Y, four-strength control + *B. subtilis* K424.

## Data Availability

Data are contained within the article.
